# TMT-Based Quantitative Proteomic Analysis Reveals the Effect of Bone Marrow Derived Mesenchymal Stem Cell on Hair Follicle Regeneration

**DOI:** 10.3389/fphar.2021.658040

**Published:** 2021-06-14

**Authors:** Chao Zhang, YuanHong Li, Jie Qin, ChengQian Yu, Gang Ma, HongDuo Chen, XueGang Xu

**Affiliations:** ^1^ Department of Dermatology, The First Hospital of China Medical University, Shenyang, China; ^2^ NHC Key Laboratory of Immunodermatology (China Medical University), Shenyang, China; ^3^ Key Laboratory of Immunodermatology (China Medical University), Ministry of Education, Shenyang, China; ^4^ Key Laboratory for the Genetics of Developmental and Neuropsychiatric Disorders (Ministry of Education), Bio-X Institutes, Shanghai Jiao Tong University, Shanghai, China

**Keywords:** bone marrow-derived mesenchymal stem cell, hair follicle stem cell, hair follicle regeneration, TMT-based quantitative proteomics analysis, parallel reaction monitoring

## Abstract

Hair loss (HL) is a common chronic problem of poorly defined etiology. Herein, we explored the functionality of bone marrow-derived mesenchymal stem cell (BMSC) and conditioned medium (MSC-CM) as regulators of hair follicle proliferation and regeneration, and the mechanistic basis for such activity. BMSC were cultured and identified *in vitro* through the induction of multilineage differentiation and the use of a CCK-8 kit. The dorsal skin of mice was then injected with BMSC and MSC-CM, and the impact of these injections on hair cycle transition and hair follicle stem cell (HFSC) proliferation was then evaluated via hematoxylin and eosin (H&E) staining and immunofluorescent (IF) staining. We then conducted a tandem mass tags (TMT)-based quantitative proteomic analysis of control mice and mice treated with BMSC or MSC-CM to identify differentially expressed proteins (DEPs) associated with these treatments. Parallel reaction monitoring (PRM) was utilized as a means of verifying our proteomic analysis results. Herein, we found that BMSC and MSC-CM injection resulted in the transition of telogen hair follicles to anagen hair follicles, and we observed the enhanced proliferation of HFSCs positive for Krt15 and Sox9. Our TMT analyses identified 1,060 and 770 DEPs (fold change＞1.2 or＜0.83 and *p* ＜ 0.05) when comparing the BMSC vs. control and MSC-CM vs. control groups, respectively. Subsequent PRM validation of 14 selected DEPs confirmed these findings, and led to the identification of Stmn1, Ncapd2, Krt25, and Ctps1 as hub DEPs in a protein-protein interaction network. Together, these data suggest that BMSC and MSC-CM treatment can promote the proliferation of HFSCs, thereby facilitating hair follicle regeneration. Our proteomics analyses further indicate that Krt25, Cpm, Stmn1, and Mb may play central roles in hair follicle transition in this context and may represent viable clinical targets for the treatment of HL.

## Introduction

Hair loss (HL) is a common cosmetic condition that can have a significant adverse impact on the quality of life (QOL) and well-being of affected individuals. Hair follicles function as miniature organs, and are generally relatively synchronous, undergoing three primary stages of growth: a growth (anagen) phase, a rest (telogen) phase, and an apoptosis-mediated regression (catagen) stage (Paus, 1998). Aside from scar tissue- or wound-induced alopecia, the majority of HL-related disorders are attributable to aberrant hair follicle morphology or dysregulating cycling, including the shortening of the anagen stage or the prolongation of the telogen stage (Cotsarelis and Millar, 2001; Varothai and Bergfeld, 2014).

Hair follicle stem cells (HFSCs) are essential for the regeneration of hair follicles and the transition of these follicles from the telogen to anagen stage. These HFSCs exist as a small cellular subpopulation within the midportion of the follicle at the arrector pili muscle attachment site (Cotsarelis et al., 1990; Blanpain et al., 2004), and can be identified at the molecular level owing to their staining for Krt15(Lyle et al., 1998; Liu et al., 2003) and Sox9 (Vidal et al., 2005; Nowak et al., 2008; Chacón-Martínez et al., 2017).

Bone marrow-derived mesenchymal stem cells (BMSC) are multipotent stem cells (Friedenstein et al., 1974), and they can be readily obtained, grown, and expanded, all while exhibiting minimal immunogenicity (Le Blanc et al., 2003; Bara et al., 2014). Importantly, BMSC can also differentiate into diverse cell types *in vitro* (Prockop, 1997; Pittenger et al., 1999). These properties make BMSC promising candidates for use in the context of regenerative medicine and other tissue engineering applications (Bianco et al., 2001; Lane et al., 2014).

To date, BMSC and conditioned medium (MSC-CM) have been studied in-depth in the context of wound healing assays in animal skin wound model or diabetic foot ulcers, and have been shown to promote wound repair, neoangiogenesis and to accelerate re-epithelialization. Meanwhile, the regeneration of cutaneous appendages, such as hair follicles and sebaceous glands, were also found. The ability and mechanism of BMSC and MSC-CM to induce the regeneration of hair follicles in mice intradermal injection model, however, remains to be studied in detail.

While genomic and transcriptomic analyses can provide insight into the mechanistic basis for hair follicle regeneration, such mRNA-level data may not align with true phenotypic findings given that protein expression levels are determined by multiple factors including mRNA stability, mRNA localization, protein degradation, and posttranslational modifications. Detailed proteomic analyses are therefore essential in order to fully understand the mechanisms whereby BMSC and MSC-CM influence the growth of hair follicles. Tandem mass spectrometry (MS/MS)-based approaches have emerged as a reliable approach to accurately quantifying relative protein levels in complex samples. The use of isotopomer labels to perform tandem mass tag (TMT) MS/MS analyses is increasingly common (Thompson et al., 2003), offering key advantages including high sensitivity, good reproducibility, a high signal-to-noize ratio, and the ability to multiplex up to 11 samples.

Herein, we intradermally transplanted BMSC and MSC-CM into mice and then evaluated hair cycle transition, hair follicle regeneration, and HFSC phenotypes. We additionally employed a TMT-based proteomics approach coupled with PRM to better identify key hair regeneration-related protein targets in this therapeutic context.

## Materials and Methods

### BMSC Isolation and Culture

Female C57BL/6 mice (4–6 weeks old; Beijing Vital River Laboratory Animal Technology Co., Ltd., China) were euthanized, and BMSC were isolated from the femurs of these animals. After isolation, BMSC were cultured in complete DMEM (Sigma-Aldrich, D6046) containing 10% fetal bovine serum (FBS) (Sigma-Aldrich, F2442), 2 mM L-glutamine, and penicillin/streptomycin at 37°C in 5% humidified CO_2_. Cells were grown until 80–90% confluent at which time they were passaged. Cells were passaged three times for use in downstream analyses. Supernatants were collected from cells on the fifth day of the third-passage culture, and were used as conditioned media for cell-free MSC-CM assays.

### BMSC *in vitro* Differentiation

BMSC were induced to undergo osteogenesis by treating them with low-glucose DMEM containing 10% FBS, 50 μg/ml ascorbic acid, 100 nM dexamethasone 10 mM β-glycerophosphate, and penicillin/streptomycin. Osteogenic differentiation was assessed by measuring alkaline phosphatase (ALP) activity through an azo coupling approach on day 7 following induction, and via Alizarin red staining on day 21 following induction.

Adipogenesis was induced by treating BMSC with low-glucose DMEM containing 10% FBS, 500 mM 1-methyl-3-isobutylxanthine, 1 μM dexamethasone, 200 μM indomethacin, and 10 μM insulin. After three days, this media was exchanged for adipogenic maintenance medium and cells were cultured for one additional day. This process was repeated thrice, after which oil red O staining was performed to evaluate lipid accumulation within these cells.

### CCK-8 Assay

A CCK-8 kit was used to gage BMSC proliferation. Briefly, cells were plated in 96-well plates (2000/well in 100 µL), and 10 μL of CCK8 reagent was added per well. Plates were then incubated for 2 h at 37°C, after which absorbance (OD) at 450 nm was measured, and growth curves were established by plotting time against OD values.

### Animals

Female C57BL/6 mice (18–20 g, 7 weeks old, Beijing Vital River Laboratory Animal Technology Co., Ltd.) were used for all *in vivo* experiments, which were conducted in accordance with the China Medical University Guidelines for the Care and Use of Laboratory Animals.

### Intradermal Injections

Hair clippers and an electric razor were used to remove hair from the dorsal flank of all 60 mice, which were subsequently randomized into three treatment groups (*n* = 20 each): a control group, an MSC-CM group, and a BMSC group. Control mice were administered media containing no cells, while mice in the MSC-CM group were administered cell-free MSC-CM, and mice in the BMSC group were administered 1 × 10^6^ BMSC in fresh CM. A 250 μL total injection volume was used for a mouse, with 16 separate intradermal injections being made on the dorsal skin of treated mice. One injection was made every other day for two weeks in total.

### Histological Analyses

On days 0, 7, 10, and 15 after the initial injection, mice in each group were euthanized and samples of full-thickness dorsal skin were collected, fixed for 24 h with 4% paraformaldehyde (PFA), and paraffin-embedded prior to slicing into 5 μm sections that were subjected to hematoxylin and eosin staining (H&E) based upon standard protocols.

Hair follicle length in each section was quantified by randomly selecting three fields of view per section and measuring the follicle length from papilla to epidermis with the ImageJ program. A total of 20 follicles were measured per mouse, with five mice per group being analyzed at each time point.

### Immunofluorescent Staining

The paraffin-embedded 5 μm-thick sections prepared above were used for IF staining. Antigen retrieval was first achieved by treating samples for 4 min with citrate buffer at 100°C, followed by blocking for 1 h with 5% BSA at room temperature. Sections were then probed overnight with primary rabbit anti-Krt15 (1:100 dilution, Abcam, ab52816) and anti-Ki67 (1:100 dilution, Novus Biologicals, NBP2-22112) or anti-Sox9 (1:250 dilution, Abcam, ab185966) and anti-Ki67 (1:100 dilution, Novus Biologicals, NBP2-22112) at 4°C, followed by incubation for 1 h with goat anti-rabbit IgG conjugated to AF594 (1:500 dilution, Cell Signaling, #8889) or goat anti-mouse IgG conjugated to AF488 (1:200 dilution, Abcam, ab150113) at room temperature. DAPI was then used to stain cell nuclei, and a Fluoview FV1000 confocal laser scanning fluorescence microscope (Olympus, Tokyo, Japan) was employed to image cells.

### Protein Extraction, Trypsin Digestion, and TMT Proteomic Labeling

A total of four full-thickness dorsal skin samples from each group on day 7 post-injection were collected and subjected to total protein extraction with the MinuteTM Total Protein Extraction Kit for Skin Tissue (Invent Biotechnologies, lnc. Beijing, China). Trypsin digestion was then performed by reducing the protein lysate with a 5 mM dithiothreitol (DTT) solution for 30 min at 56°C, followed by alkylation with 11 mM iodoacetamide (IAA) for 15 min at room temperature protected from light. Next, 200 mM tetraethylammonium bromide (TEAB) was used to dilute samples to a urea concentration of <2 M, and trypsin was added at a 1:50 trypsin-to-protein mass ratio overnight at 37°C, followed by a second digestion for 4 h at a 1:100 trypsin-to-protein mass ratio. Once digestion was complete, a Strata X C18 SPE column (Phenomenex, CA, United States) was used to achieve protein desalting, and samples were vacuum-dried. These peptides were then resuspended in 0.5 M TEAB and processed with a 6-plex TMT kit (Thermo Fisher Scientific, CA, United States) based on provided directions. Briefly, one unit of TMT reagent, which was required to label 100 μg of peptide was thawed and reconstituted in acetonitrile (ACN). The peptide mixtures were then incubated with the prepared TMT reagent for 2 h at room temperature. Finally, TMT-labeled peptide mixtures were pooled, desalted and dried by vacuum centrifugation.

### HPLC Fractionation, LC-MS/MS Analysis, and Database Search

TMT-labeled peptides were fractionated via high pH reverse-phase high-performance liquid chromatography (HPLC) with an Agilent 300Extend C18 column (5 μm particles, 4.6 mm ID, 250 mm long). Briefly, TMT-labeled peptide mixture was first separated into 60 fractions with a gradient of 8–32% acetonitrile (ACN, pH 9.0) over 60 min. Then, the fractions were combined into nine fractionated simplified samples and dried by vacuum centrifuging.

Liquid chromatography-tandem mass spectrometry (LC-MS/MS) was performed at PTM Biolab Hangzhou (Hangzhou, China). All fragments were dissolved in solvent A (0.1% formic acid and 2% acetonitrile), directly loaded onto a home-made reversed-phase analytical column (15 cm length, 75 μm i. d.). The gradient was comprised of an increase from 6 to 23% solvent B (0.1% formic acid in 100% acetonitrile) over 42 min, 23–35% solvent B in 12 min and climbing to 80% solvent B in 3 min. Finally, a holding phase at 80% solvent B for the last 3 min was performed, all at a constant flow rate of 450 nL/min on a NanoElute ultraperformance liquid chromatography (UPLC) system. The peptides were subjected to Capillary source followed by tandem mass spectrometry (MS/MS) in tims-TOF Pro coupled online to the UPLC. The electrospray voltage applied was 2.0 kV and intact peptides were detected in the TOF. The secondary mass spectrometry scan range was from 100 to 1700 m/z. Data collection was acquired in parallel accumulation-serial fragmentation (PASEF) mode. A first mass spectrometry was collected and PASEF mode was used for 10 times to collect the secondary mass spectrometry with the charge of the parent ion in the range of 0–5. The dynamic exclusion was set at 30 s to avoid repeated scanning of parent ions.

The Maxquant search engine (v.1.6.6.0) was used to analyze the resultant data, with MS/MS spectra being searched against the *Mus*_musculus_10,090 database (17,045 sequences) concatenated with a reverse decoy database. Trypsin/*p* was specified as cleavage enzyme allowing up to two missing cleavages. The mass tolerance for precursor ions was set as 20 ppm in First search and 5 ppm in Main search, respectively, and 0.02 Da was set for fragment ions. Carbamidomethyl on Cys was specified as fixed modification and oxidation on Met was specified as variable modifications. FDR was adjusted to <1% and minimum score for peptides was set >40.

### Parallel Reaction Monitoring Analyses

Protein isolation and trypsinization were conducted as above, after which a PRM mass spectrometric analysis was performed via MS/MS with Q Exactive Plus (Thermo) coupled online to the UPLC. The LC parameters, electrospray voltage, scan range, and Orbitrap resolution were identical to those used for TMT analyses. Automatic gain control was set to 3E6 for full MS and 1E5 for MS/MS, with a maximum IT of 20 ms for full MS and auto for MS/MS. An MS/MS isolation window of 2.0 m/z was used, and the resultant MS data were analyzed with Skyline (v.3.6). Peptide settings: enzyme was set as Trypsin [KR/P], Max missed cleavage set as 2. The peptide length was set as 8–25, Variable modification was set as Carbamidomethyl on Cys and oxidation on Met, and max variable modifications was set as 3. Transition settings: precursor charges were set as 2, 3, ion charges were set as 1, 2, ion types were set as b, y, *p*. The product ions were set as from ion three to last ion, the ion match tolerance was set as 0.02 Da.

### Bioinformatics Analyses

DEPs were subjected to functional annotation based upon Gene Ontology (GO) classifications, subcellular localization, and COG/KOG categories, using the UniProt-GOA database and InterProScan, Wolfpsort, and the COG/KOG database.

The enrichment of DEPs for particular GO terms, KEGG pathways, and protein domains were assessed using the GO annotations, KEGG, and InterPro databases, respectively, using two-tailed Fisher’s exact test with a *p* < 0.05 as the threshold of significance.

A protein-protein interaction (PPI) network incorporating identified DEPs was prepared with the STRING database (https://string-db.org), using a score >0.4 as the significance threshold for identified interactions.

### Statistical Analysis

Data are means ± SD from three or more experiments, and were compared via one- or two-way ANOVAs with Tukey’s test. *p* < 0.05 was the significance threshold.

## Results

### BMSC Characterization

**FIGURE 1:**
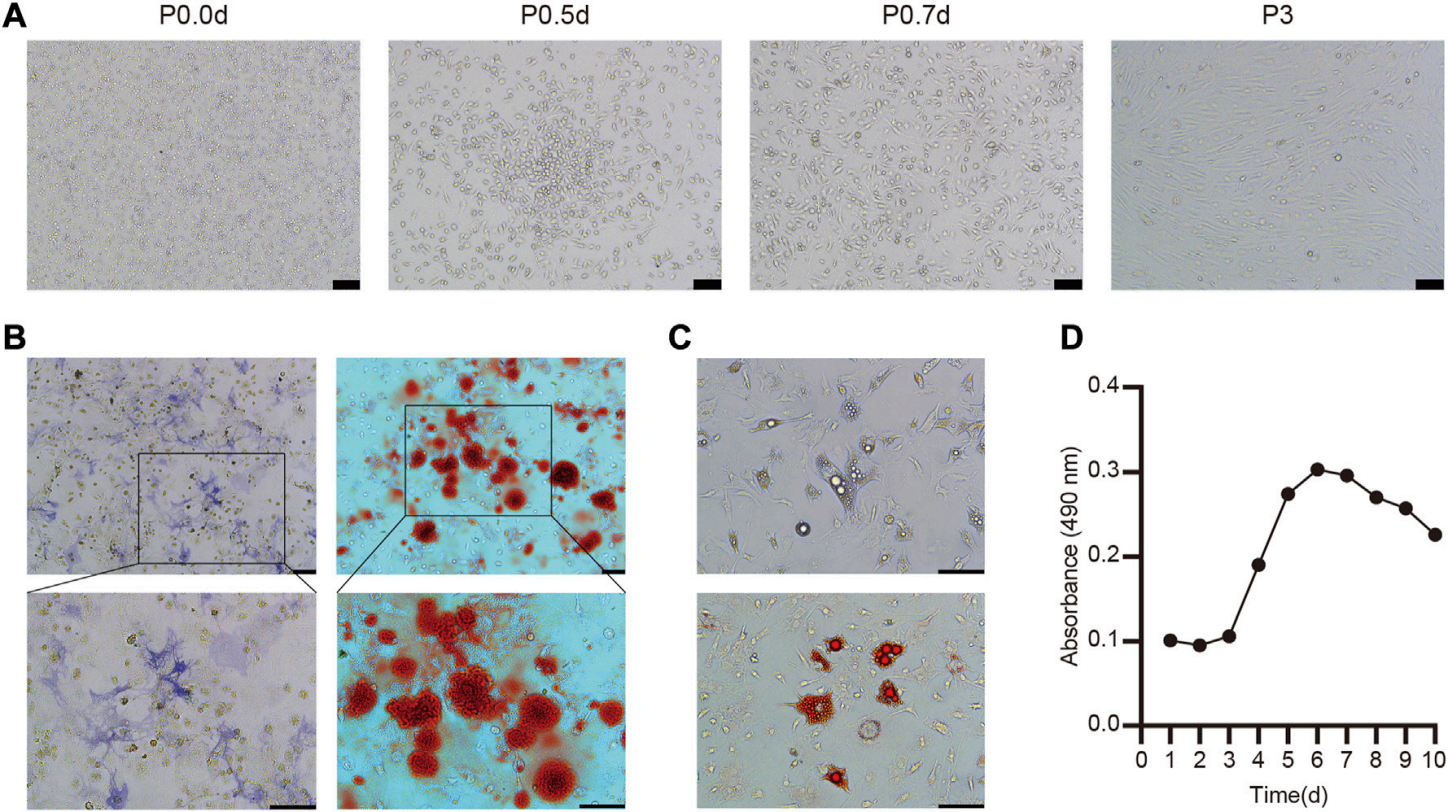
Culture, identification and biological characteristics of BMSC **(A)** The morphology of primary and the third-passage BMSC from C57BL/6 mice was observed under light microscopy **(B)** Osteogenic differentiation was stained with ALP and Alizarin red **(C)** Adipogenic differentiation of BMSC and stained with oil red O **(D)** Cell growth curve of the third passage BMSC.

Cultured BMSC exhibit a normal round morphology when cultured at 10^6^ cells/mL. Within 24–48 h of isolation, these cells began to adhere and form radial colonies that were evidence within 5 days, growing to 80–90% confluence within 7 days. At the end of this 7-day period, cells exhibited polygonal, triangular, and spindle-like morphology, and were subsequently passaged. Remaining erythrocytes and other non-adherent cells were removed by repeatedly changing the media and passaging these cells, and BMSC from the third passage exhibited uniform fibroblast-like spindle-shaped morphology and were considered suitable for *in vivo* use (Figure 1A).

To explore the ability of these BMSC to undergo osteogenesis *in vitro,* they were treated with osteogenic differentiation medium and ALP staining was conducted after 7 days via the azo coupling approach, revealing positively stained (blue-purple) cytoplasmic precipitates. Furthermore, Alizarin red staining was used to confirm the differentiation of these cells based on the presence of calcium nodules and bone mineralization on day 21 (Figure 1B). We also determined that these BMSC were able to differentiate into adipocytes, as evidenced by the presence of lipid droplets within 4 days and the presence of clear lipid vacuoles upon oil red O staining on day 12 of the differentiation process (Figure 1C).

Overall, these findings thus confirmed that these isolated cells were functionally and morphologically consistent with BMSC, making them ideal for use in our experimental model system.

### BMSC Growth Kinetics

A CCK-8 assay was next used to evaluate the growth kinetics of third-passage BMSC, revealing a standard “S”-shaped curve with a lag phase from days 1–3, followed by logarithmic growth from days 3–6, a plateau beginning on day 6, and a slight decrease in cell numbers beginning on day 8 (Figure 1D). Based upon this curve, we elected to utilize third-passaged BMSC collected on the fifth day of culture for all intradermal injection experiments.

### The Impact of BMSC and MSC-CM on Hair Follicle Transition *in vivo*

**FIGURE 2:**
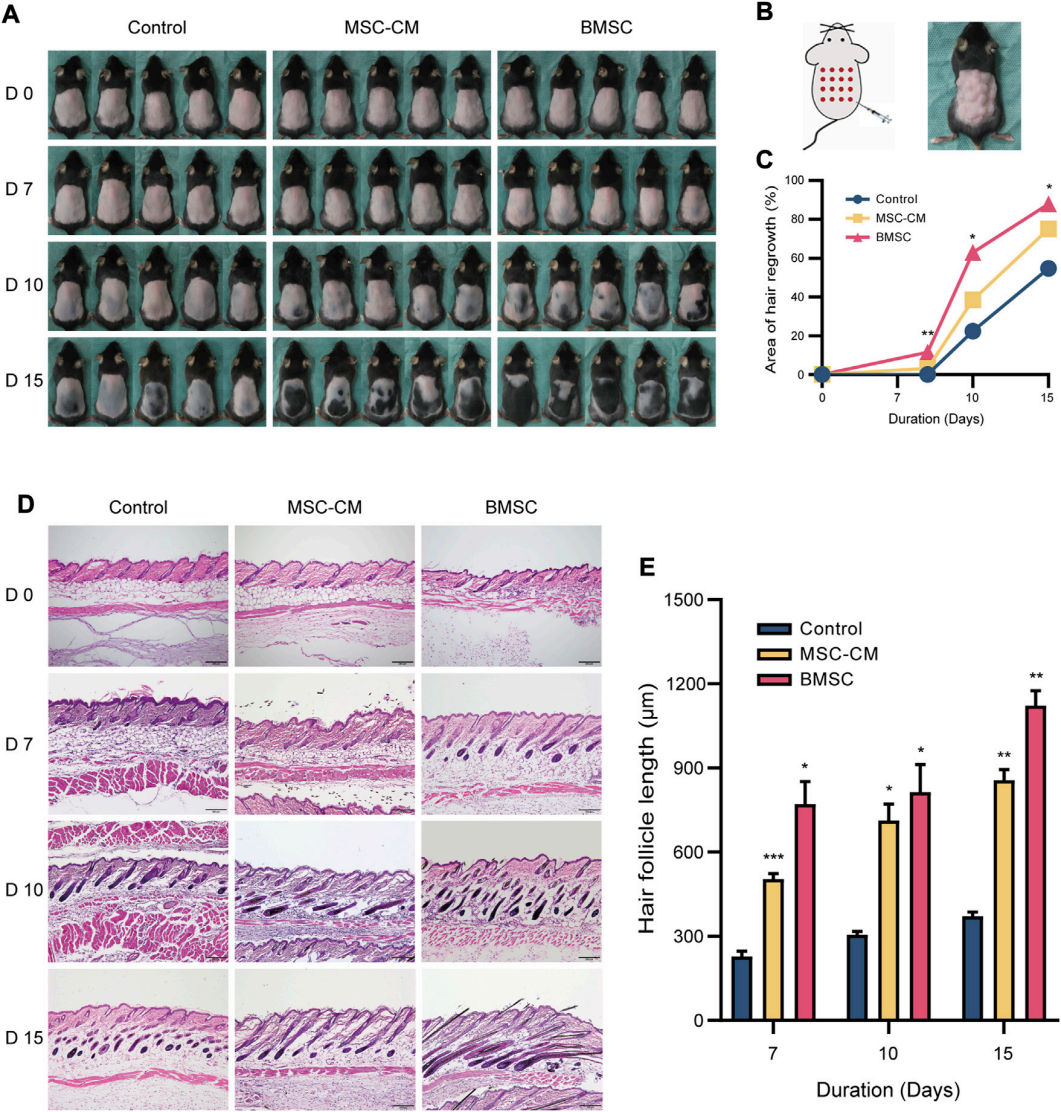
Macroscopic and histologic assessment of mice on 0, 7, 10, 15 days after intradermally injection of MSC-CM and BMSC **(A)** The dorsal skin of control, MSC-CM treated and BMSC treated mice was photographed on 0, 7, 10, and 15 days **(B)** Schematic representation of injection sites and photos of mouse after injection **(C)** Quantification of area of hair regrowth. values are represented in percentage. *p < 0.05, **p < 0.01 **(D)** Representative H&E staining images of dorsal skin sections on 0, 7, 10, and 15 days for determination of hair follicle cycle and length. Scale bar: 200 µm **(E)** Graph represents length of hair follicle of the visible microscopic field (at-least three fields) with 20 measurements was taken in all three groups. Values are represented in μm. Data are expressed as mean ± SD. *p < 0.05, **p < 0.01, ***p < 0.001.

For C57BL/6 mice, shaved skin typically appears pink during the telogen stage but darkens upon anagen initiation (Sato et al., 1999). All mice appeared to be in the telogen phase prior to injection. On day 7 post-injection, however, dark punctate spots were evident in the skin of mice in the BMSC and MSC-CM treatment groups but not in control animals. On day 10 post-injection, the majority of mice in the BMSC and MSC-CM treatment groups had entered the anagen phase, with the tips of hair shafts having begun to emerge from the epidermal layer and with dorsal pigmentation having increased substantially in both groups, with some darkening beginning to appear in the control group as well. On day 15 post-injection, hair growth was nearly complete in the BMSC group, and the majority of the dorsal skin of MSC-CM-treated mice was dark, whereas in control mice this pigmentation and hair growth was sporadic and uneven (Figure 2A). The schematic representation of injection sites and photos of mouse after injection were shown in Figure 2B. Together, these data revealed that BMSC and MSC-CM treatment were sufficient to induce hair cycle transition in mice. Quantification of these data confirmed that BMSC treatment significantly enhanced hair growth relative to control treatment on days 7, 10, and 15 post-treatment (*p < 0.05, **p < 0.01) (Figure 2C).

### Histopathological Staining

We next conducted the H&E staining of tissue samples from these mice, which revealed that samples of control, MSC-CM and BMSC groups were all in telogen on day 0. On day 7 post-injection, control group samples were still in the telogen phase, whereas BMSC- and MSC-CM-treated samples had entered the anagen phase and exhibited increased numbers of hair matrix cells. On day 10 post-injection, control group samples exhibited an increase in the volume of the hair follicle dermal papilla, while the dermal papilla in the MSC-CM-treated samples was largely surrounded by hair matrix cells, and was surrounded by dermal papilla in the BMSC-treated samples with clear evidence of hair shaft development. On day 15 post-injection, most follicles in the control group had entered the anagen phase, while there was a clear increase in hair follicle density in the BMSC group and the BMSC-CM group (Figure 2D). Quantitative analyses confirmed that hair follicle length increased over time after injection, with significantly longer hair follicles being observed in the BMSC and MSC-CM treatment groups relative to the control group (*p < 0.05, **p < 0.01, ***p < 0.001) (Figure 2E).

### BMSC and MSC-CM Treatments Induce HFSC Activation

**FIGURE 3:**
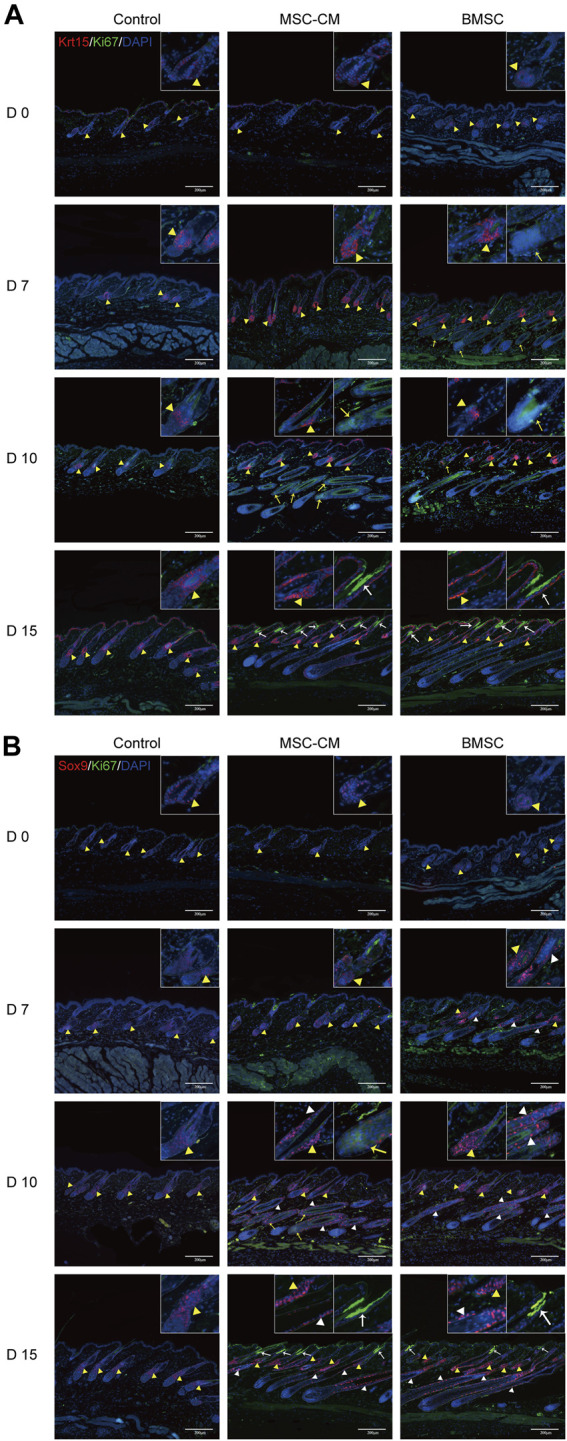
Proliferation of hair follicle stem cells after BMSC and MSC-CM treatment **(A)** Krt15 (red fluorescence) was observed in the Bu region and Ki67 (green fluorescence) was observed in the bulb area and infundibulum of hair follicle at 0, 7, 10 and 15 days **(B)** Sox9 fluorescence (red fluorescence) in the Bu and ORS region and Ki67 (green fluorescence) in the bulb area and infundibulum at 0, 7, 10 and 15 days. Nuclei were stained with DAPI (blue). The dashed line delineates the hair follicle structure. The yellow triangles show the expression site of the bulge (Bu) region and white triangles show the expression site of outer root sheaths (ORS). The bulb areas are indicated by yellow arrows and infundibula are indicated by white arrows. Scale bar: 200 μm.

HFSCs are key mediators of hair regeneration, and their ability to proliferate is closely linked to hair follicle transition and overall hair growth. As such, we next analyzed the expression of the HFSC markers Krt15 and Sox9 in the dorsal skin of our treated mice via IF staining. This approach revealed that there was no difference in the fluorescence intensity between three groups on days 0 and there were significantly more Krt15 + proliferating cells in the bulge region and Ki67 + proliferating cells in the bulb area and infundibulum of hair follicles in the BMSC and MSC-CM treatment groups on days 7, 10, and 15 of treatment relative to the control group (Figure 3A). We also detected a significant increase in Sox9 expression in the bulge and outer root sheath (ORS) region and Ki67 expression in the bulb area and infundibulum of follicles from BMSC- and MSC-CM-treated mice (Figure 3B). Together, these data show that treatment with BMSC or MSC-CM can trigger HFSC activation and thereby promote hair regrowth.

### Identification of Proteins Differentially Expressed in Response to BMSC and MSC-CM Treatment

We next conducted a TMT labeling-based proteomic analysis of dorsal skin samples from mice in our control, BMSC, and MSC-CM treatment groups. In total, we identified 63,131 peptides, of which 60,598 were unique, leading to the identification of 5,046 quantifiable proteins. Those proteins with a fold change (FC) > 1.30 or <0.77 and *p* < 0.05 when comparing the BMSC or MSC-CM groups to the control group were identified as differentially expressed proteins (DEPs).

**FIGURE 4:**
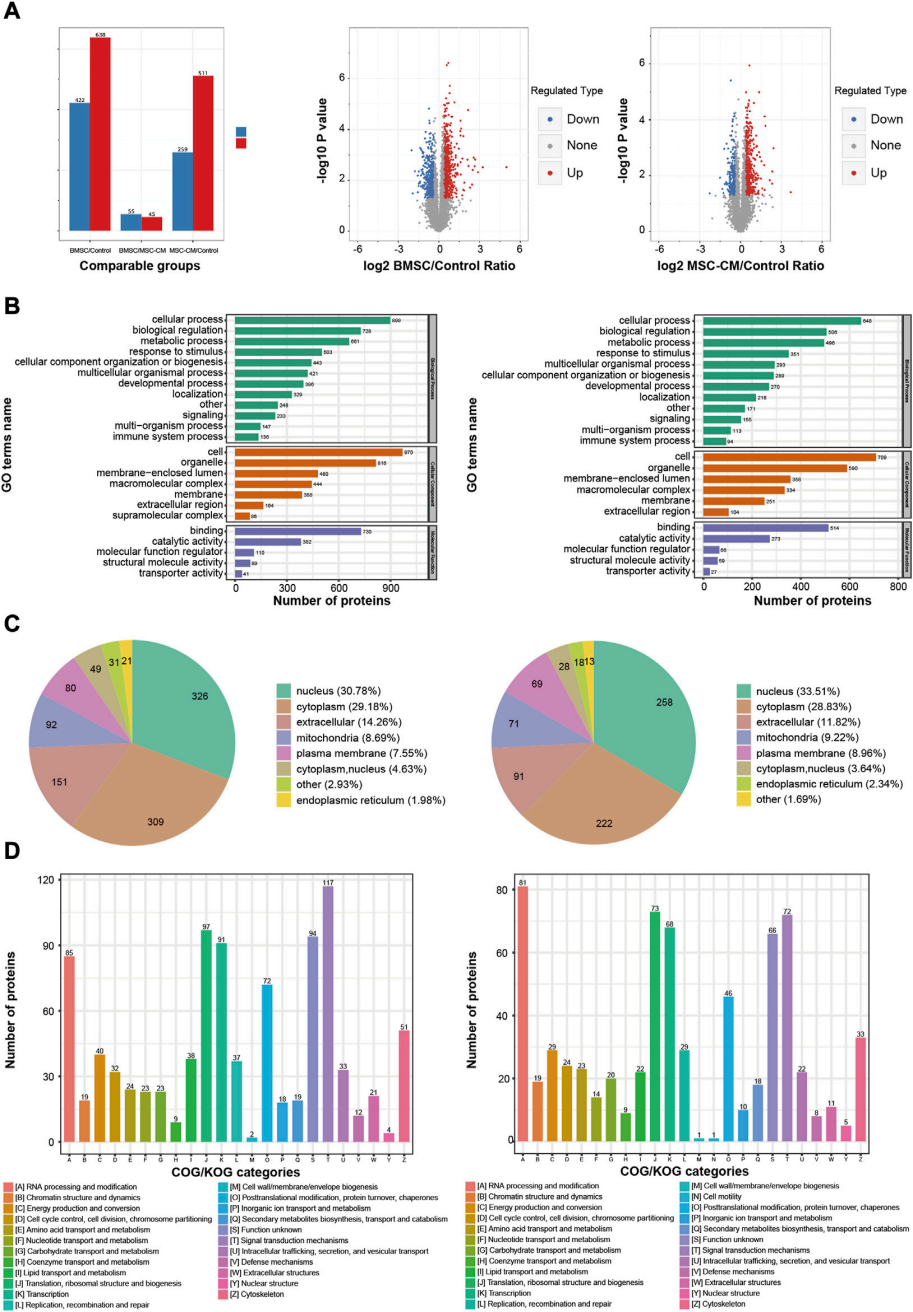
Functional annotations and classification of the DEPs in BMSC/Control group and MSC-CM/Control group **(A)** Statistics and volcano plots of the DEPs **(B)** Statistical distribution chart of the DEPs under each GO category (second Level) **(C)** Subcellular localization chart of the DEPs **(D)** COG/KOG functional classification chart of the DEPs. Note: DEPs: Differentially Expressed Proteins.

In total, we identified 1,060 DEPs when comparing the BMSC and control group samples (638 upregulated, 422 downregulated), while 770 were identified when comparing the MSC-CM and control group samples (511 upregulated, 259 downregulated) (Figure 4A).

In the BMSC vs. control and MSC-CM vs. control groups, overall DEPs were classified based upon their enrichment in specific GO biological process (BP), molecular function (MF), and cellular component (CC) terms. With respect to BPs, DEPs were primarily involved in cellular processes (899 proteins; 648 proteins), biological regulation (728 proteins; 506 proteins), and metabolic processes (661 proteins; 496 proteins). With respect to CCs, these DEPs were primarily enriched in cell (970 proteins; 709 proteins), organelle (816 proteins; 590 proteins), and membrane-enclosed lumen (480 proteins; 358 proteins). With respect to MFs, DEPs were mainly enriched for binding (730 proteins; 514 proteins), catalytic activity (382 proteins; 273 proteins), and molecular function regulation (110 proteins; 66 proteins) (Figure 4B).

Wolfpsort was used to predict the subcellular localization of these DEPs, which were primarily localized to the nucleus (30.78%; 33.51%), cytoplasm (29.18%; 28.83%), and extracellular compartment (14.26%; 11.82%) (Figure 4C).

COG/KOG functional classification analyses assigned these DEPs to 23 functional KOG classifications that were primarily associated with signal transduction (117 proteins; 72 proteins), translation, ribosomal structure, and biogenesis (97 proteins; 73 proteins), and transcription (91 proteins; 68 proteins) (Figure 4D).

### Functional Enrichment Analyses of Treatment-Related DEPs

To better understand the relationship between identified DEPs and hair regrowth, we next conducted functional enrichment analyses of proteins that were differentially expressed between the BMSC or MSC-CM groups and the control group based upon GO, KEGG pathway, and protein domain analyses.

**FIGURE 5:**
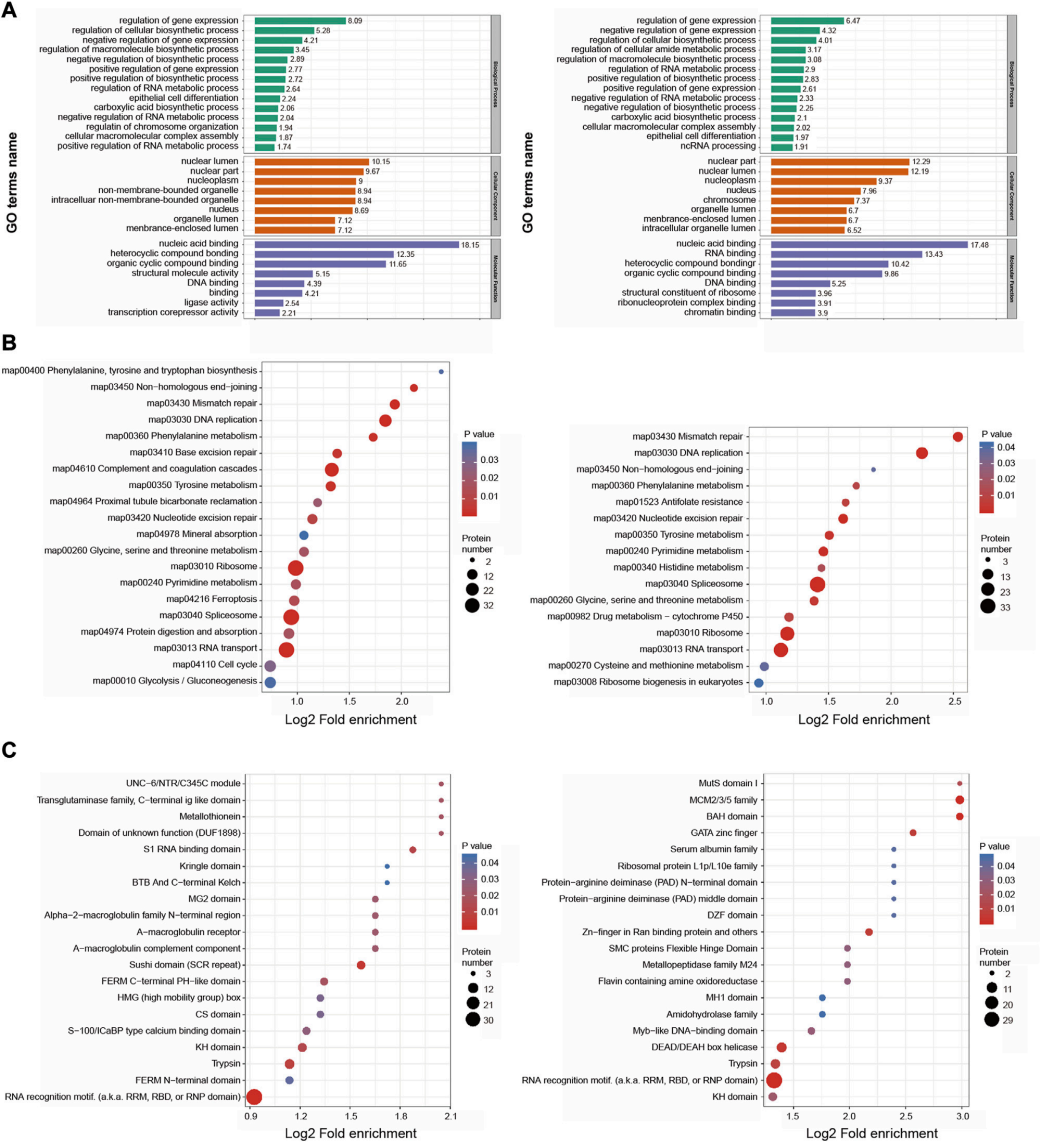
Functional enrichment analysis of the DEPs in BMSC/Control group and MSC-CM/Control group **(A)** GO enrichment analysis of the DEPs were annotated and enriched by biological process, cell component and molecular function **(B)** KEGG pathway enrichment analysis of the DEPs **(C)** Protein domain enrichment analysis of the DEPs.

With respect to GO biological processes, DEPs were enriched in the regulation of gene expression, regulation of cellular biosynthetic processes, and negative regulation of gene expression. They were additionally enriched for cellular components including the nuclear lumen, nuclear part, and nucleoplasm, and for molecular functions including nucleic acid binding, heterocyclic compound binding, and organic cyclic compound binding (Figure 5A).

KEGG pathway enrichment analyses revealed these DEPs to be enriched in DNA replication, mismatch repair, splicesome, and ribosome pathways (Figure 5B).

Protein domain enrichment analyses revealed these DEPs to be enriched in RNA recognition motifs and trypsin domain (Figure 5C).

Together, based on functional classifications and enrichment analyses, these DEPs are involved in genetic material replication, cell cycle control, and metabolic regulation, which are related to hair cycle transition.

### PRM Validation

**FIGURE 6:**
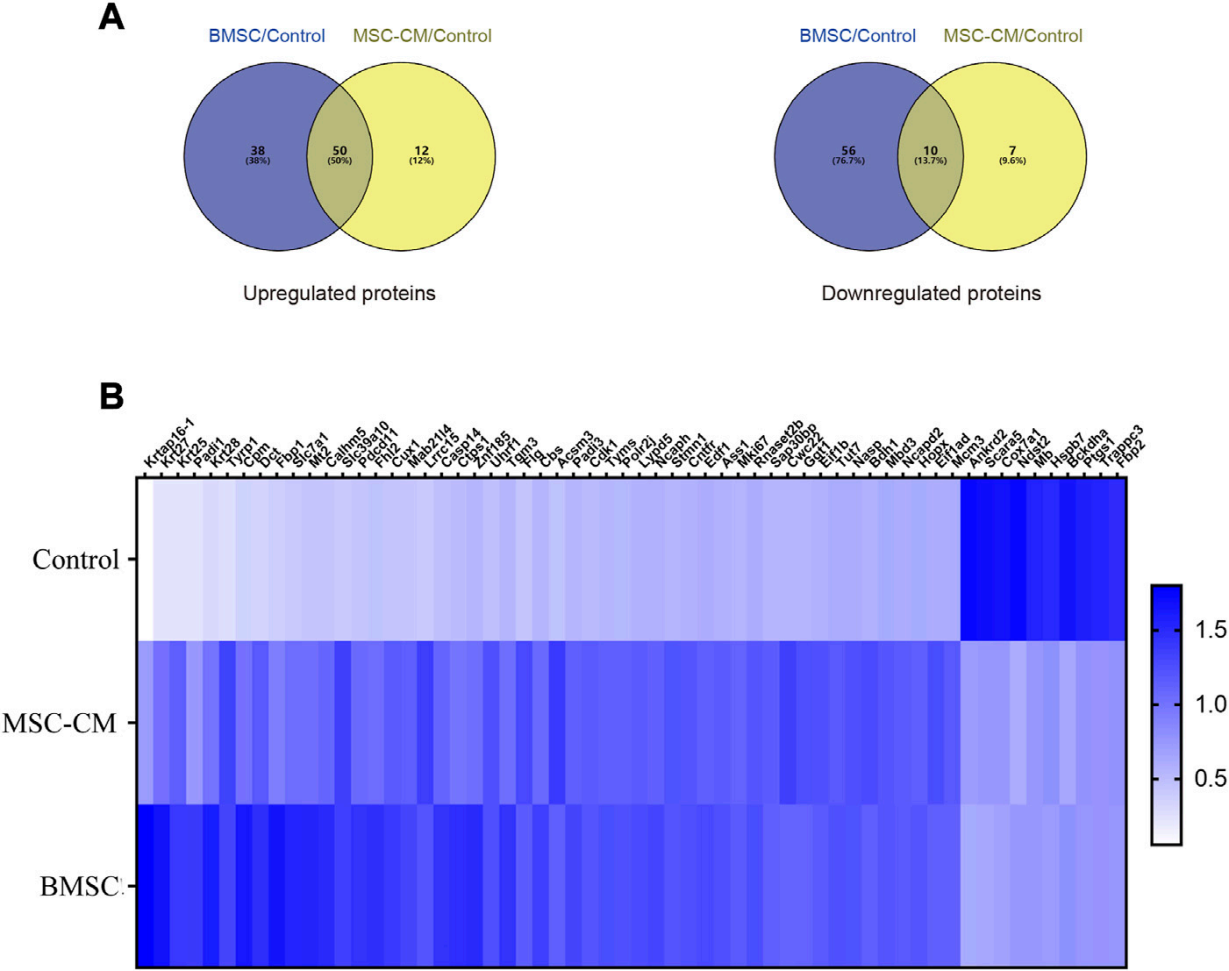
Discovery of candidate hair follicle regeneration related proteins **(A)** Venn diagrams of differentially expressed proteins between BMSC vs. control and MSC-CM vs. control groups. Fold change >1.85 or <0.54 and *p*-value < 0.05 **(B)** Heatmap of the 60 optimized DEPs in the Control, MSC-CM, and BMSC groups. The X-coordinate stands for the DEPs, while Y-coordinate stands for the different groups.

**FIGURE 7:**
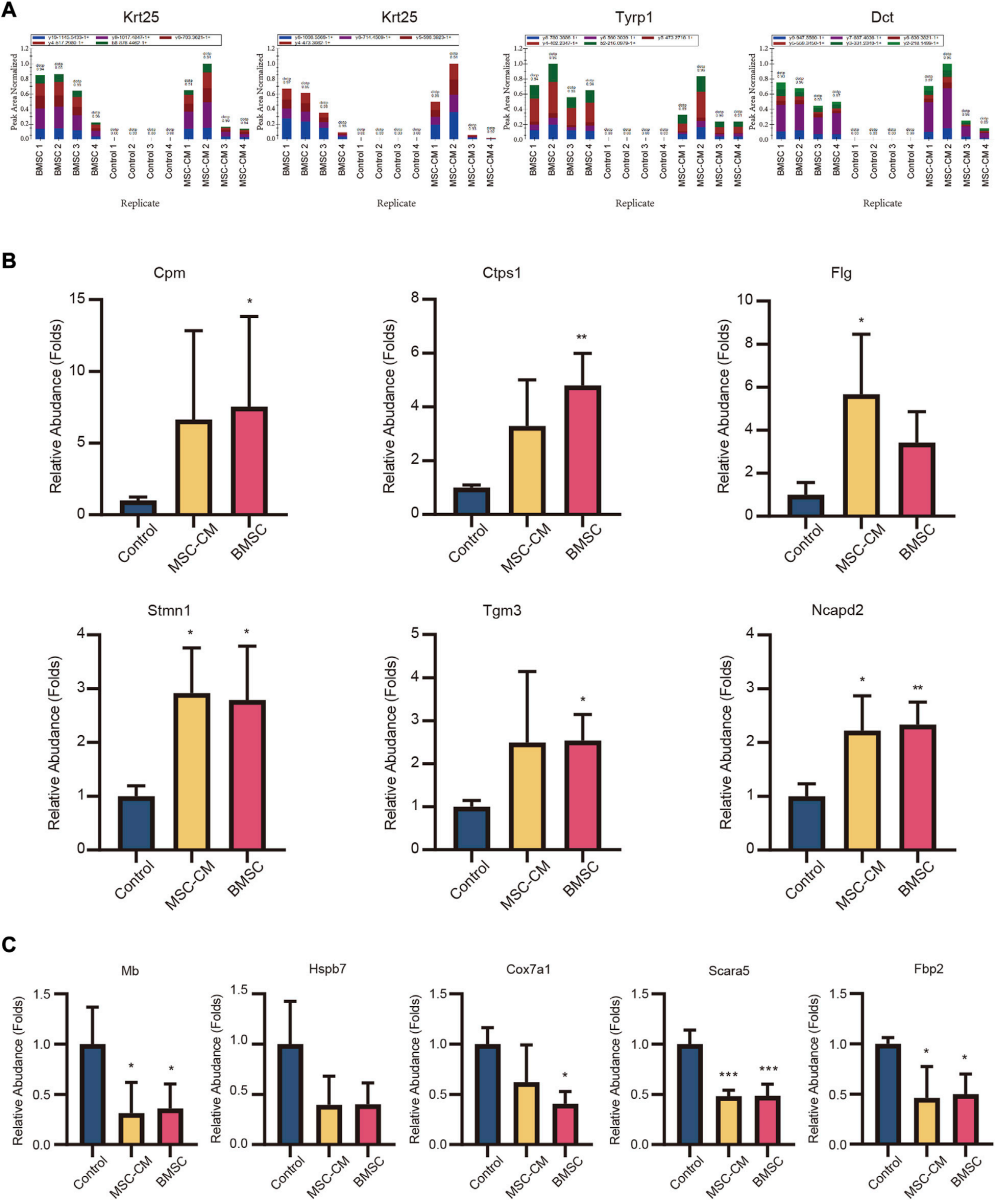
Validation of candidate hair follicle regeneration related proteins. Parallel reaction monitoring (PRM) of nine upregulated proteins **(A)** (Krt25, Tyrp1, Dct) **(B)** (Cpm, Ctps1, Flg, Stmn1, Tgm3, Ncapd2) and **(C)** five downregulated proteins (Hspb7, Mb, Cox7a1, Fbp2, Scara5).

DEPs that were shared between the BMSC vs. control and MSC-CM vs. control datasets (FC > 1.85 or <0.54 and *p* < 0.05) were identified using Venn diagrams, leading to the identification of 60 optimized DEPs, of which 50 and 10 were up- and down-regulated, respectively (Figure 6A; Tables 1, 2). These proteins are shown in a heatmap in Figure 6B. Of these 60 DEPs, we selected nine upregulated DEPs (Krt25, Cpm, Ctps1, Flg, Ncapd2, Tyrp1, Dct, Tgm3, Stmn1) (Figures 7A,B) and five downregulated DEPs (Mb, Hspb7, Cox7a1, Scara5, Fbp2) (Figure 7C) for subsequent PRM validation. Our quantitative PRM results confirmed that these candidate DEPs exhibited trends comparable to those observed upon TMT analysis, confirming the reliability of our proteomics data (Table 3).

**TABLE 1:** The 50 optimized up-regulated expressed proteins.

Protein ID	Protein name	Gene name	Fold change (BMSCs/control)	*p*-value (BMSCs/control)	Fold change (mscs-cm/control)	*p*-value (mscs-cm/control)
A2A5X5	Keratin-associated protein 16–1	Krtap16–1	31.561	3.03E-03	12.86	3.92E-02
Q9Z320	Keratin, type I cytoskeletal 27	Krt27	7.373	4.80E-03	4.436	1.46E-02
Q8VCW2	Keratin, type I cytoskeletal 25	Krt25	6.192	2.77E-03	5.027	7.05E-03
Q9Z185	Protein-arginine deiminase type-1	Padi1	5.635	3.89E-03	5.357	1.04E-03
A6BLY7	Keratin, type I cytoskeletal 28	Krt28	5.455	1.45E-02	3.406	3.25E-02
P07147	5,6-Dihydroxyindole-2-carboxylic acid oxidase	Tyrp1	4.899	1.91E-02	5.007	1.92E-02
Q80V42	Carboxypeptidase M	Cpm	4.572	2.74E-03	2.833	1.26E-02
P29812	L-dopachrome tautomerase	Dct	4.457	1.73E-05	3.564	1.64E-04
Q9QXD6	Fructose-1,6-bisphosphatase 1	Fbp1	4.31	1.53E-03	2.421	4.01E-02
Q09143	High affinity cationic amino acid transporter 1	Slc7a1	3.674	1.24E-02	2.487	3.50E-02
P02798	Metallothionein-2	Mt2	3.479	1.81E-04	2.281	8.23E-03
Q8R100	Calcium homeostasis modulator protein 5	Calhm5	3.469	2.35E-02	2.506	4.53E-02
Q6P5F6	Zinc transporter ZIP10	Slc39a10	3.342	1.39E-03	3.357	4.21E-03
Q6NS46	Protein RRP5 homolog	Pdcd11	3.295	3.84E-02	2.426	9.72E-03
O70433	Four and a half LIM domains protein 2	Fhl2	3.118	9.26E-05	2.224	9.31E-04
P53564	Homeobox protein cut-like 1	Cux1	3.062	5.87E-03	2.587	1.01E-05
Q8CEZ4	Protein mab-21-like 4	Mab21l4	3.039	1.93E-03	2.685	2.16E-03
Q80 × 72	Leucine-rich repeat-containing protein 15	Lrrc15	3.02	4.49E-05	3.42	7.61E-05
O89094	Caspase-14	Casp14	3.016	1.71E-04	2.303	1.84E-03
P70698	CTP synthase 1	Ctps1	2.984	1.16E-04	1.981	2.51E-02
Q62394	Zinc finger protein 185	Znf185	2.764	5.70E-04	1.883	1.09E-02
Q8VDF2	E3 ubiquitin-protein ligase UHRF1	Uhrf1	2.619	2.69E-03	2.57	1.46E-03
Q08189	Protein-glutamine gamma-glutamyltransferase E	Tgm3	2.614	5.94E-03	1.854	3.75E-02
P11088	Filaggrin (fragment)	Flg	2.587	5.12E-03	2.896	3.02E-03
Q91WT9	Cystathionine beta-synthase	Cbs	2.47	5.79E-03	1.931	3.65E-02
Q3UNX5	Acyl-coenzyme a synthetase ACSM3, mitochondrial	Acsm3	2.459	4.44E-02	3.024	2.87E-02
Q9Z184	Protein-arginine deiminase type-3	Padi3	2.384	2.05E-02	2.078	1.38E-02
P11440	Cyclin-dependent kinase 1	Cdk1	2.359	1.04E-03	2.308	8.70E-04
P07607	Thymidylate synthase	Tyms	2.327	6.90E-03	2.11	2.64E-02
O08740	DNA-directed RNA polymerase II subunit RPB11	Polr2j	2.298	7.26E-03	2.125	8.61E-03
Q9D7Z7	Ly6/PLAUR domain-containing protein 5	Lypd5	2.255	3.05E-02	2.055	3.75E-02
Q8C156	Condensin complex subunit 2	Ncaph	2.211	6.54E-03	1.908	3.10E-02
P54227	Stathmin OS = *Mus musculus*	Stmn1	2.209	8.55E-06	2.219	3.69E-04
O88507	Ciliary neurotrophic factor receptor subunit alpha	Cntfr	2.207	1.66E-02	2.102	1.54E-02
Q9JMG1	Endothelial differentiation-related factor 1	Edf1	2.16	1.18E-02	1.908	2.87E-02
P16460	Argininosuccinate synthase	Ass1	2.114	2.85E-03	1.958	9.77E-03
E9PVX6	Proliferation marker protein Ki-67	Mki67	2.091	3.10E-03	2.005	4.46E-03
C0HKG6	Ribonuclease T2-B	Rnaset2b	2.081	3.82E-03	1.975	3.23E-02
Q02614	SAP30-binding protein	Sap30bp	2.076	1.55E-03	2.116	2.68E-03
Q8C5N3	Pre-mRNA-splicing factor CWC22 homolog	Cwc22	2.006	4.81E-02	2.492	1.23E-02
Q60928	Glutathione hydrolase 1 proenzyme	Ggt1	2.005	1.64E-02	2.247	1.99E-02
Q9CXU9	Eukaryotic translation initiation factor 1 b	Eif1b	2.003	2.29E-02	2.093	4.62E-02
Q5BLK4	Terminal uridylyltransferase 7	Tut7	2.002	6.58E-03	1.924	6.56E-03
Q99MD9	Nuclear autoantigenic sperm protein	Nasp	1.991	2.30E-03	2.022	2.47E-03
Q80XN0	D-beta-hydroxybutyrate dehydrogenase, mitochondrial	Bdh1	1.962	2.41E-02	2.199	1.16E-02
Q9Z2D8	Methyl-CpG-binding domain protein 3	Mbd3	1.959	1.27E-02	1.926	1.73E-02
Q8K2Z4	Condensin complex subunit 1	Ncapd2	1.951	1.92E-04	1.93	5.00E-04
Q8R1H0	Homeodomain-only protein	Hopx	1.926	1.61E-02	1.858	1.23E-02
Q3THJ3	Probable RNA-binding protein EIF1AD	Eif1ad	1.881	1.54E-02	2.096	1.14E-02
P25206	DNA replication licensing factor MCM3	Mcm3	1.858	1.51E-03	1.955	7.45E-04

**TABLE 2:** The 10 optimized down-regulated expressed proteins.

Protein ID	Protein name	Gene name	Fold change (BMSCs/control)	*p*-value (BMSCs/control)	Fold change (mscs-cm/control)	*p*-value (mscs-cm/control)
Q9WV06	Ankyrin repeat domain-containing protein 2	Ankrd2	0.358	1.33E-02	0.418	4.21E-02
Q8K299	Scavenger receptor class a member 5	Scara5	0.386	5.30E-04	0.437	1.10E-03
P56392	Cytochrome c oxidase subunit 7A1, mitochondrial	Cox7a1	0.399	1.68E-02	0.447	2.00E-02
P52850	Bifunctional heparan sulfate N-deacetylase/N-sulfotransferase 2	Ndst2	0.428	1.64E-02	0.352	1.18E-02
P04247	Myoglobin	Mb	0.473	2.13E-02	0.482	1.72E-02
P35385	Heat shock protein beta-7	Hspb7	0.487	7.50E-03	0.53	7.28E-03
P50136	2-Oxoisovalerate dehydrogenase subunit alpha, mitochondrial	Bckdha	0.489	4.80E-02	0.396	3.00E-02
P22437	Prostaglandin G/H synthase 1	Ptgs1	0.49	2.22E-02	0.499	1.93E-02
O55013	Trafficking protein particle complex subunit 3	Trappc3	0.503	3.45E-02	0.499	2.09E-02
P70695	Fructose-1,6-bisphosphatase isozyme 2	Fbp2	0.51	1.29E-03	0.531	3.51E-03

**TABLE 3:** PRM analysis of 14 candidate proteins.

Protein ID	Prorein name	Gene name	Peptide sequence	BMSCs/Control Ratio	BMSCs/Control ratio (TMT)	MSCs-CM/Control Ratio	MSCs-CM/Control Ratio (TMT)
Q8VCW2	Keratin, type I cytoskeletal 25	Krt25	LASYLDNVQALQEANADLEQK	∞	6.19	∞	5.03
			LEYEQLLNVK				
P07147	5,6-Dihydroxyindole-2-carboxylic acid oxidase	Tyrp1	NTVEGYSAPTGK	∞	4.90	∞	5.01
P29812	L-dopachrome tautomerase	Dct	EQFLGALDLAK	∞	4.46	∞	3.56
Q80V42	Carboxypeptidase M	Cpm	LPLFWNDNK	7.51	4.57	6.65	2.83
P70698	CTP synthase 1	Ctps1	GLGLSPDLVVCR	4.74	2.98	3.24	1.98
			NVLGWQDANSTEFDPK				
P11088	Filaggrin (fragment)	Flg	AGSSSGSGVQGASAGGLAADASR	3.42	2.59	5.66	2.90
			GQSPDASGR				
P54227	Stathmin	Stmn1	ASGQAFELILSPR	2.79	2.21	2.92	2.22
			ESVPDFPLSPPK				
Q08189	Protein-glutamine gamma-glutamyltransferase E	Tgm3	VITNFNSAHDTDR	2.87	2.61	1.80	1.85
			IAYSQYER				
Q8K2Z4	Condensin complex subunit 1	Ncapd2	HSQELSSILDDAALSGSDR	2.34	1.95	2.22	1.93
			GFAAFLTELAER				
P04247	Myoglobin	Mb	VEADLAGHGQEVLIGLFK	0.36	0.47	0.31	0.48
			GQHAAEIQPLAQSHATK				
P35385	Heat shock protein beta-7	Hspb7	CQLPEDVDPTSVTSALR	0.40	0.49	0.40	0.53
P56392	Cytochrome c oxidase subunit 7A1, mitochondrial	Cox7a1	LFQADNDLPVHLK	0.41	0.40	0.62	0.45
Q8K299	Scavenger receptor class a member 5	Scara5	LLQAPLQADLTEQVWK	0.49	0.39	0.48	0.44
			VGVLGEELADVGGALR				
P70695	Fructose-1,6-bisphosphatase isozyme 2	Fbp2	EAVITAQER	0.50	0.51	0.46	0.53
			VPLILGSPEDVQEYLSCVQR				

### PPI Network Analysis

**FIGURE 8:**
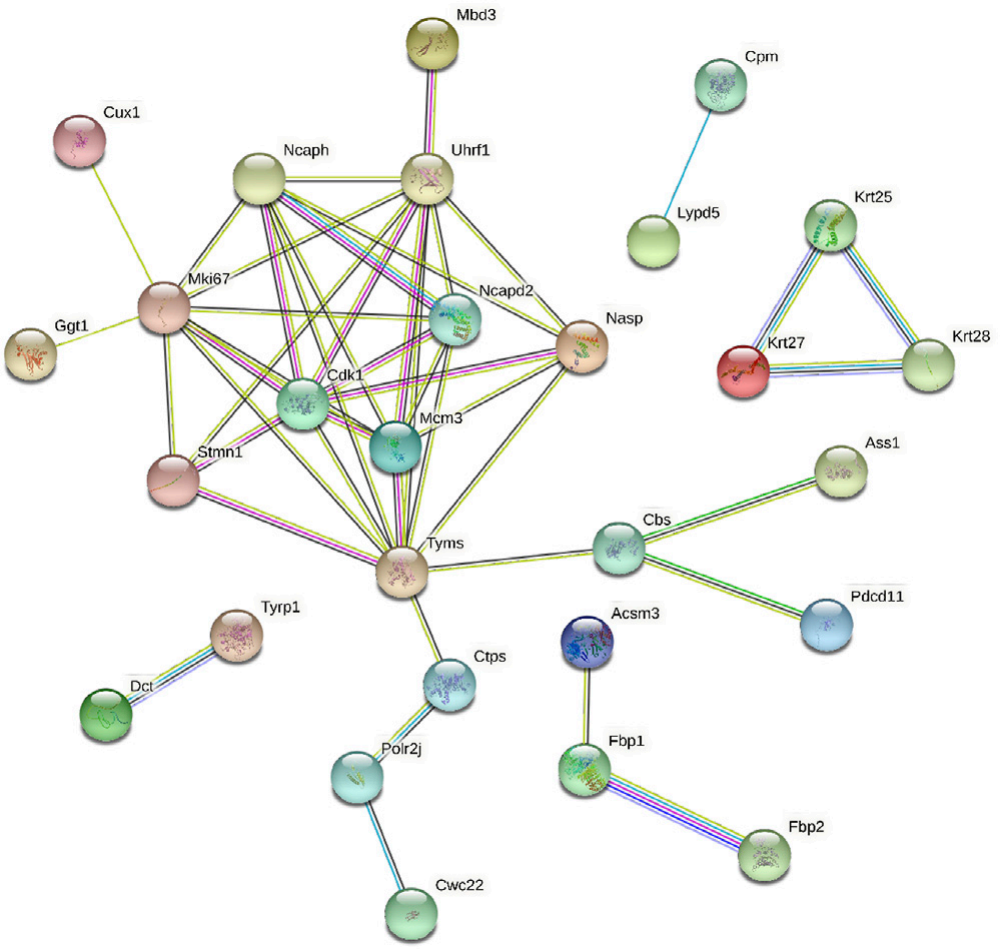
(A) PPI network of some of the optimized upregulated and downregulated proteins.

Lastly, we constructed a PPI network incorporating DEPs that were shared between the BMSC vs. control and MSC-CM vs. control datasets, including some of the proteins from our PRM validation analysis. Several of our PRM validation proteins were identified as hub proteins within this PPI network, including Stathmin 1 (Stmn1), Non-SMC condensin I complex subunit D2 (Ncapd2), Keratin, type I cytoskeletal 25 (Krt25), and Cytidine triphosphate synthetase 1 (Ctps1) (Figure 8). These proteins may thus play key roles in mitosis and migration, stem cell proliferation, hair follicle regeneration, and hair cycle transition.

## Discussion

BMSC and derivatives thereof are frequently studied as a potential resource for use in therapeutic applications as a means of repairing damaged tissue (Zhang et al., 2018), treating inflammatory diseases (Shi et al., 2018; Harrell et al., 2019), or normalizing aberrant immunological and inflammatory responses in a range of contexts. BMSC are particularly promising in these therapeutic contexts as they can undergo self-renewal, multipotent differentiation, and enable patient-specific tissue regeneration without any significant ethical concerns (Park et al., 2019).

Significant progress has been made in applying BMSC and conditioned medium as a means of treating androgenic alopecia and alopecia areata (Elmaadawi et al., 2018). BMSC-conditioned medium collected during BMSC culture has been used as a cell-free therapeutic approach in stem cell therapy. Paracrine factors including basic fibroblast growth factor (bFGF), vascular endothelial growth factor (VEGF), epidermal growth factor (EGF), hepatocyte growth factor (HGF), insulin-like growth factor-1 (IGF-1), and platelet-derived growth factor (PDGF) are present in MSC-CM preparations and are likely to contribute to tissue follicle regeneration (Fu et al., 2019).

The mechanisms whereby BMSC can facilitate hair regeneration, however, remain poorly understood. As such, in the present study, we injected BMSC and MSC-CM into the dorsal side of 7-week-old C57BL/6 mice and explored the biological basis of BMSC-induced hair regrowth. We confirmed that both of these treatment approaches were able to expedite the telogen-to-anagen transition necessary to enhance hair growth.

Hair loss can be classified as being either permanent, cicatricial, non-reversible scarring alopecia, or temporary, non-cicatricial, reversible non-scarring alopecia. In cases of scarring alopecia, as in patients with cutaneous lupus erythematosus and lichen planus, inflammation leads to irreversible HFSC loss. In contrast, forms of non-scarring alopecia such as alopecia areata (AA) and androgenic alopecia (AGA) are characterized by the loss of progenitor cells but the preservation of HFSCs, allowing for the restoration of hair growth (Garza et al., 2011). Promoting the proliferation and differentiation of HFSCs is thus necessary to facilitate hair regeneration and telogen-to-anagen transition. Several signaling pathways regulate HFSCs activation and they include Wnt/β-catenin (Deschene et al., 2014), BMP, and mTOR (Deng et al., 2015) signaling pathways.

HFSCs are activated via a two-step process during hair regeneration: Secondary hair germ cells are first activated by dermal papilla at anagen onset, followed by bulge HFSC during the anagen III phase (Greco et al., 2009). Cytokeratin 15 (Krt15) is an HFSC marker protein (Lyle et al., 1998; Liu et al., 2003), and is used together with CD200 and CD34 to monitor the efficacy of AA and AGA treatment (Elmaadawi et al., 2018). Sox9 (Vidal et al., 2005; Nowak et al., 2008) is also a potential HFSC marker. Herein, we found that the levels of Krt15-and Sox9-positive cells rose in bulge and ORS regions following BMSC and MSC-CM injection, confirming that these treatments were able to induce telogen-to-anagen hair cycling via promoting HFSC proliferation.

We also found that BMSC and MSC-CM treatment were associated with significant increases in the expression of certain key hair structure- and induction-related proteins. For example, keratins, which encode intermediate filaments, are involved in several key processes. The type I keratin gene Krt25 is expressed in the hair medulla and all three layers of the inner root sheath (IRS) (Langbein et al., 2006), wherein it is involved in protecting, supporting, and molding the hair shaft. Ansar (Ansar et al., 2015) and Zernov (Zernov et al., 2016) et al. found that Krt25 mutations were damaging to intermediate filament formation and hair follicle development, resulting in autosomal recessive hypotrichosis. Moreover, the transglutaminase (TGase) isoform TGase-3 is expressed within the cuticle and cortex of growing hair fibers wherein it is involved in progressive hair shaft scaffolding via driving the formation of isopeptide bonds between intermediate filaments and keratin-associated proteins (KAPs). Tarsca et al. have also shown this enzyme to play a role in regulating mouse anagen hair fiber rigidity (Tarcsa et al., 1997), and Sebastien et al. have referred to is as a possible mediator in the context of hair shaft scaffolding (Thibaut et al., 2009). Susan et al. also showed that hair lacking TGase-3 was thinner and that there were clear cuticle cell alterations in mice lacking TGase-3 (John et al., 2012). We also found the expression of the hair melanin biosynthetic proteins 5,6-dihydroxyindole-2-carboxylic acid oxidase (Tyrp1) (Zdarsky et al., 1990; Kobayashi et al., 1994) and L-dopachrome tautomerase (Dct) (Jackson et al., 1992; Tsukamoto et al., 1992) to be significantly increased in skin samples following BMSC and MSC-CM treatment.

Many studies have shown T cells and macrophages to be recruited and to induce hair regeneration via the activation of HFSC differentiation (Rahmani et al., 2020) during physiologic hair cycling (Castellana et al., 2014; Ali et al., 2017), wounding, and depilation-induced hair growth (Chen et al., 2015; Lee et al., 2017; Wang et al., 2017; Rahmani et al., 2018). Notably, one of our validated DEPs identified in this study, carboxypeptidase M (Cpm), is often used as a marker for the differentiation of monocytes into macrophages (Rehli et al., 1995; Krause et al., 1998). We observed significant Cpm upregulation following BMSC and MSC-CM injection, suggesting that such treatment may be involved in macrophage recruitment and consequent anagen onset. We also identified cytidine triphosphate (CTP) synthetase 1 (Ctps1) to be significantly upregulated in this experimental system, which is noteworthy given that it is involved in activated T cell proliferation (Martin et al., 2014). Our data thus suggested that BMSC and MSC-CM treatment induce hair follicle regeneration through mechanisms associated with immunomodulation.

We additionally identified multiple upregulated proteins related to the cell cycle and proliferation. Filaggrin (Flg), for example, is involved in mechanical barrier function in healthy skin, with Salerno et al. having shown melanoma cells to upregulate Flg (Salerno et al., 2016). Leick et al. also successfully knocked out Flg in DM93 human melanoma cells via a CRISPR/Cas9 approach and thereby clarified its role in the context of cellular growth (Leick et al., 2019). Stathmin 1 (Stmn1) is a protein involved in destabilizing microtubules that it is involved in mitosis and migration by controlling free tubulin dimer availability within cells (Ringhoff and Cassimeris, 2009). Stmn1 depletion has been shown to contribute to cell cycle arrest (Chen et al., 2007) and enhanced apoptotic death (Alli et al., 2007; Ma et al., 2017). Bichsel et al. also previously demonstrated that Stmn1 deletion promoted expedited catagen transition and prematurely suppressed follicular proliferation, suggesting it plays a critical role in hair follicle cycling (Bichsel et al., 2016). Non-SMC condensin I complex subunit D2 (Ncapd2) is a condensin I complex subunit encoded on chromosome 12p13.3 that is primarily involved in chromosome condensation and segregation. Zhang et al. determined that Ncapd2 is an essential mediator of cell cycle progression and triple-negative breast cancer (TNBC) cell migration such that when it was knocked down, TNBC cells failed to proliferate or exhibit invasive activity, and instead underwent apoptotic death (Zhang et al., 2020).

In addition to these upregulated proteins, we also validated multiple proteins that were downregulated in the BMSC and MSC-CM groups relative to control mice (Hspb7, Mb, Cox7a1, Fbp2, Scara5). Hspb7, Mb, Cox7a1, Fbp2, and Scara5 have all previously been identified as tumor suppressor genes (Naderi, 2018) in breast cancer (Braganza et al., 2019), non-small cell lung cancer (Zhao et al., 2019), gastric cancer (Li et al., 2013), sarcoma (Huangyang et al., 2020), and hepatocellular carcinoma (Huang et al., 2010), wherein they can suppress tumor cell invasion, proliferation, and migration. In contrast, our data suggest that BMSC and MSC-CM treatment can enhance proliferation and hair follicle regeneration via reducing the expression of these key proteins.

## Conclusion

In summary, we herein evaluated the ability of BMSC and MSC-CM to promote the regeneration of hair follicles. We found that both of these treatments were capable of promoting follicle telogen-to-anagen transition in C57BL/6 mice, increasing follicle length and driving HFSC proliferation. Our TMT-based proteomics approach identified Krt25, Cpm, Stmn1, and Mb as candidate follicle regeneration-related proteins, and these findings were validated via PRM. Overall, these data thus suggest that BMSC and MSC-CM may represent ideal therapeutic approaches to anagen induction and hair growth stimulation as a means of treating HL.

## Data Availability

The datasets presented in this study can be found in online repositories. The names of the repository/repositories and accession number(s) can be found in the article/Supplementary material.
