# Supplementary material for: TMT-Based Quantitative Proteomic Analysis Reveals the Effect of Bone Marrow Derived Mesenchymal Stem Cell on Hair Follicle Regeneration
Source: Front Pharmacol. 2021 Jun 14;12:658040. doi: 10.3389/fphar.2021.658040 (PMC8237093; doi:10.3389/fphar.2021.658040)
Supplement: Supplementary file 1 [file datasheet2.docx]

Supplementary Material

# Supplementary Data

The mass spectrometry proteomics data have been deposited to the PRIDE Archive (http://www.ebi.ac.uk/pride/archive/) via the PRIDE partner repository with the data set identifier PXD022845

Username: [reviewer_pxd022845@ebi.ac.uk](mailto:reviewer_pxd022845@ebi.ac.uk)

Password: Zf79qQA4

Supplementary file: Protein expression and annotations of differentially expressed proteins in TMT-based quantitative proteomic analysis. (fold change> 1.30 or < 0.77 and P < 0.05). xlsx

DOI: 10.6084/m9.figshare.13635236
